# Violence risk and personality assessment in adolescents by Structured Assessment of Violence Risk in Youth (SAVRY) and high school personality questionnaire (HSPQ): Focus on protective factors strengthening

**DOI:** 10.3389/fpsyt.2022.1067450

**Published:** 2023-01-10

**Authors:** Petra Skřivánková, Marek Páv, Karolína Faberová, Derek Perkins, Hedvika Boukalová, David Adam, Aneta Mazouchová, Ilona Gillernová, Martin Anders, Eva Kitzlerová

**Affiliations:** ^1^Department of Psychiatry, Bohnice Psychiatric Hospital, Prague, Czechia; ^2^Department of Psychiatry, First Faculty of Medicine Charles University in Prague, General University Hospital in Prague, Prague, Czechia; ^3^Department of Psychology, Faculty of Arts, Charles University, Prague, Czechia; ^4^Department of Law and Criminology, Royal Holloway University of London, Egham, United Kingdom; ^5^West London Mental Health NHS Trust, Broadmoor Hospital, London, United Kingdom; ^6^Police Presidium of the Czech Republic, Prague, Czechia; ^7^Department of Psychiatry and Medical Psychology, Third Faculty of Medicine Charles University, Prague, Czechia; ^8^Department of Economic Statistics, University of Economics and Business, Prague, Czechia

**Keywords:** violence risk assessment, protective factor, high school personality questionnaire (HSPQ), juvenile offender, factor analysis, Structured Assessment of Violence Risk in Youth (SAVRY)

## Abstract

**Background:**

Adolescents are most at risk of engaging in violent interaction. Targeting violence risk and protective factors is essential for correctly understanding and assessing their role in potential violence. We aimed to use the Structured Assessment of Violence Risk in Youth (SAVRY) tool within the sample of adolescents to capture violence risk and protective factors and personality variables related to risk and protective factors. We further aimed to identify which violence risk and protective factors were positively or negatively related to violence within personal history and if any personality traits are typical for violent and non-violent adolescents. Identifying broader or underlying constructs within the SAVRY tool factor analysis can enable appropriate therapeutic targeting.

**Methods:**

We used the Czech standardized version of the SAVRY tool. The study sample comprised 175 men and 226 women aged 12–18 years divided into two categories according to the presence or absence of violence in their personal history. Mann-Whitney *U* test was used to compare numerical variables between the two groups. SAVRY factor analysis with varimax rotation was used to determine the item factors. We administered the High School Personality Questionnaire (HSPQ) to capture adolescents’ personality characteristics.

**Results:**

In our sample, there were 151 participants with violence in their personal histories and 250 non-violent participants. Non-violent adolescents had higher values for all six SAVRY protective factors. The strongest protective factor was P3, Strong attachment and bonds across gender or a history of violence. Using factor analysis, we identified three SAVRY internal factors: social conduct, assimilation, and maladaptation. The SAVRY protective factors were significantly positively related to several factors in the HSPQ questionnaire.

**Conclusion:**

The results highlight the significance of protective factors and their relationship with violence prevalence. HSPQ diagnostics could be helpful in clinically targeting personality-based violence risks and protective factors. The therapeutic focus should be on tension, peer rejection, and anxiety. It is also essential to foster positive attitudes toward authority, prosocial behavior, and attitudes toward school. These strategies can help strengthen protective factors of the SAVRY.

## 1. Introduction

Various violence assessment tools adhering to the risk-need-responsivity model have been developed in the past decades to guide professionals in violence risk evaluation, therapeutic planning, and legal decision-making (1–4). The concept of violence protective factors has received more attention in the past decade, revealing its role in desistance from offending (5, 6). Therefore, there is more widespread use of assessments including both risk and protective factors, either in the form of complementary tools (to structured professional Judgment tools-SPJ, such as HCR-20 (Historical Clinical Risk Management-20)–SAPROF (Structured Assessment of Protective Factors) or a single tool, for example, Structured Assessment of Violence Risk in Youth (SAVRY) (5, 7). Protective factor assessment in children and adolescents is potentially even more important than in adults, as it can positively influence individual development using strength-oriented treatment approaches (8). In addition, to find proper resource allocations, it is necessary to identify intervention targets that can reduce the emotional, social, and economic costs of life-course-persistent offending (9–11).

Adolescence is a period of complex psychosocial and biological changes associated with the completion of sexual maturation and the establishment of individual identity (12, 13). As an individual faces an ego identity crisis, role confusion can occur, and within a peer group, this can lead to antisocial and risky behaviors such as lying or substance abuse (14). Violent behavior can also occur as a quick means of getting a reward, or due to the inability to correctly interpret social situations (15–17), or to attain peer appraisal (18). Exposure to violence is considered a risk factor for future violent behavior (19, 20). Boys are more susceptible to violent action, with a higher violent and antisocial behavior rate than girls (21). The main risk factors for adolescent violent behavior development are early adverse social context, gross, and inconsistent parenting at an early age, early behavioral problems, lack of social, and cognitive maturity at school, failure at school, lack of parental supervision during adolescence, and contact with problematic peers (22, 23). A dynamic cascade model of serious violence in adolescence is proposed, which links the above risk factors to a developmental model that begins with the birth of a child into a socially unfavorable environment. This subsequently increases stress within the family and negatively affects early parental competencies (22, 24). Children from exclusionary environments had a significantly higher prevalence of dissociative symptoms, and heredity played a significant role in children growing up in disturbed environments, affecting aggressive behavior and environmental rule-breaking (25).

As adolescent personality development continues, psychological assessment of personality is used to identify emerging traits and coping mechanisms related to violent behavior in various settings, such as custody evaluations (26, 27). The relationship between personality traits and violence has been studied extensively; violent behavior-related traits, including manipulativeness, inability to take responsibility for one’s actions, and tendencies to rationalize are often identified (28, 29). In adolescents, low self-esteem, emotional dysregulation, or impaired impulse control are predictors of violence (30–32). In addition, adolescents with a high level of Cluster B personality disorder symptoms (including antisocial and borderline personalities) are likely to commit violent acts during adolescence and early adulthood (28). The use of alcohol as a coping mechanism acts as a disinhibitory factor, increasing the likelihood of violence (33). A lower IQ is also associated with violence perpetration (24, 34, 35).

While there is a relatively deep understanding of how risk factors are reflected in the prevalence of violent behavior, there is much less understanding of which factors in an adolescent’s personality or environment reduce the risk of violent behavior. The essential violence protective factors in adolescence are above-average intelligence, positive relationships with the family and school, and a positive relationship with at least one parent (8, 36). Notably, minimal exposure to physical punishment, future orientation, future goals, rejection of violence in peer relationships and interest groups, and no ADHD diagnosis were observed (8). One of the tools that employs protective factors is the SAVRY scale, which includes both risk and protective factors (8). An alternative for SAVRY use, especially within younger adolescent populations, is the SAPROF-YV scale (37, 38) or SAPROF scale, capturing only protective factors, intended to complement structured professional judgment risk assessment tools, such as HCR-20 (39, 40). Several findings have demonstrated that the SAVRY has good inter-rater reliability (41, 42). It also has good predictive validity for general, violent, and non-violent offenses at different follow-up times (38, 43). This was also replicated in Czechia, as our SAVRY adaptations had an AUC of 0.865 predictive validity (24, 42). There is demonstrated higher predictive validity for SAVRY in self-reported delinquency compared to the Youth Level of Service/Case Management Inventory (YLS/CMI) and the Psychopathy Checklist: Youth Version (PCL: YV) (42). Moreover, there is evidence that the SAVRY is more sensitive to change than the VRS-YV (Violence Risk Scale-Youth Version) or SAPROF-YV (44). Notably, the overall protective SAVRY domain was related to reoffence, buffering the effects of some of the risk domains (24, 45). All 24 factors, including the specific protective factors of the SAVRY, are listed in the Section “2.2 Tools and 3 Results” below.

In the current study, we aimed to describe the prevalence of violence risk and protective factors in a sample of Czech adolescents. Second, we aimed to identify which violence risk and protective factors were positively or negatively related to violence within personal history and if any personality traits are typical for violent and non-violent adolescents. Third, we aimed to conduct factor analysis to identify factor clusters in the SAVRY tool, as its purpose is data reduction and simplification (46). Identifying broader or underlying constructs within the SAVRY tool can enable appropriate therapeutic targeting of the areas that fall into each cluster. As previous findings differ in the number and composition of these principal factors (47, 48), we decided to repeat this analysis with our sample. Last aim was to explore, whether HSPQ factors relate to the SAVRY protective factors.

## 2. Materials and methods

### 2.1. Data collection and sampling

We collected data for this study from medical, school, and social facilities across the Czech Republic. We contacted individual institutions (social and school facilities child psychiatry, outpatient clinic of child clinical psychology, children’s homes, diagnostic and educational institutions, and schools) with participation offers. Approximately one-third of the surveyed facilities (22) agreed with the research conditions, and nominated professionals were willing to participate in the study. There was no reimbursement for professional staff or participants.

Data collection in participating facilities took 15 months (1 June 2018–30 September 2019). Professionals in participating facilities engaged in the study were acquainted with the study protocol, the requirements for signing the study consent, and the research methodology. These professionals then nominated suitable probands for interviews by one of the three principal evaluators. The first author is an experienced clinical psychologist who realized most of the SAVRY ratings (310 participants), trained by The International Organization of Forensic Practitioners (TIOFP) in the SAVRY assessment. The rest of the dataset was collected by a psychologist with expertise in child psychology and a psychology student trained on the SAVRY scale. All raters received training in the SAVRY administration.

The SAVRY rating is based on semi-structured interviews. The interview was conducted with the proband and a person familiar with the case (parent, clinical worker, psychologist, or social worker). The probands and close adults were interviewed separately. When it was not possible for the principal investigator to conduct an in-person semi-structured interview with the adolescent, it was sent in paper form so that the information was available for evaluating the SAVRY scale. Data collection was then realized by professionals in direct care (mostly psychologists of the local institutions) in isolated cases by other professional staff, such as directors of children’s homes. In such cases, the test administration was preceded by a review of the relevant documentation. This did not interfere with the evaluation of the SAVRY.

Additionally, within the interviews, a primary clinical-psychological observation method was used, which helped complete the comprehensive picture of the assessed adolescents and their social environment. During the semi-structured interview, clinical observation of the adolescent and their behavior was performed, such as observing signs of nervousness (redness in the face, biting fingernails, playing with fingers) or noting psychomotor restlessness as a possible manifestation of ADHD.

The medical records were available within the health facilities for all cases, and further documents were in their files, such as forensic expert reports and court orders. This information was amended based on the information obtained from the facility’s professional staff.

Participation was offered to several hundred adolescents, pre-selected by direct-care staff as eligible for study participation and fulfilling the inclusion criteria: age 12–18 years, intelligence within lower band average or higher, and mental state enabling a valid completion of psychodiagnostic tools. The participants were then enrolled in the study. We obtained the research group (*n* = 401) of adolescents aged 12–18 divided into two subgroups according to the incidence of violence in their personal history. We defined violence by its legal criteria, and to be enrolled into the violent history group, the participant had to commit any of the following deeds: attempted murder, aggravated assault, minor physical assault, robbery, general endangerment, attempted rape, blackmail, and dangerous threats. The group with violence in their personal history (*n* = 151) consisted of 81 boys and 70 girls. The group without a history of violence (*n* = 250) comprised 94 boys and 156 girls.

We conducted a pilot study to evaluate the data collection process, the HSPQ questionnaire, and SAVRY questionnaire suitability. Tools were administered to 30 adolescents, and their relatives were interviewed to enable SAVRY coding. The pilot sample comprised 15 girls and 15 boys aged 12–18. 14 adolescents acted violently in their personal history, 10 of whom had committed three or more violent acts, while 16 were non-violent. Inter-rater reliability and concurrent SAVRY validity were established, and the translation of Item 9, interruption of early care, was adjusted. Data were collected from 373 adolescents (24).

### 2.2. Tools

#### 2.2.1. The SAVRY

The SAVRY assists professionals with transparent, unified, and standardized decisions on the probability of violence in adolescents. The authors researched violence and aggression in adolescents and various criminogenic theories. By including dynamogenic factors, the tool focuses on assessing violence risks and interventions to mitigate these risks (8). The scale was designed to guide professional violence risk assessment and the planning of interventions to reduce the likelihood of violence in adolescents (8). The scale contains 24 items of risk (or criminogenic) factors (see Table 1), divided into three parts. The historical scale contains ten risk factors (e.g., violence at an early age, child abuse, or the criminality of parents/caregivers and a history of self-harm or suicide attempts). The social scale contains six factors (e.g., peer delinquency, inconsistent education, and peer rejection), and the individual scale comprises eight factors (e.g., anger management problems, antisocial attitudes, or lack of empathy/insensitivity). The scale includes six protective factors (prosocial involvement, strong social support, strong attachments and bonds, positive attitudes toward interventions and authorities, strong ties to school, and resilience as a personality trait). The scale was developed for boys and girls aged 12–18 years. There are no cut-off scores or numerical assessments; each item has a three-level rating structure ranging from “0 = low” to “1 = moderate” to “2 = high.” The final evaluation of the likelihood of committing violence was based on the principle of professional structured assessment, one of the latest approaches to assessing the risk of committing violence. The expert evaluated this probability in the low, medium, or high categories. The record sheet also enables the recording of other risk and protective factors specified by the instrument.

**TABLE 1:** Structured Assessment of Violence Risk in Youth (SAVRY) factor analysis.

SAVRY Structured Assessment of Violence Risk in Youth		Factor loading		
		1 Dissocial behavior	2 Assimilation	3 Maladaptation
Historical	History of violence	**0.530**	0.163	0.340
	History of non-violent offending	**0.647**	0.416	0.286
	Early initiation of violence	**0.367**	0.056	0.307
	Past supervision/intervention failures	**0.509**	0.133	0.202
	History of self-harm or suicide attempts	**0.286**	0.172	0.265
	Exposure to violence in the home	0.126	**0.544**	0.170
	Childhood history of maltreatment	0.116	**0.703**	0.215
	Parental/caregiver criminality	0.160	**0.666**	0.240
	Early caregiver disruption	0.262	**0.730**	0.152
	Poor school achievement	0.236	0.372	**0.406**
Social/contextual	Peer delinquency	**0.688**	0.276	0.097
	Peer rejection	0.035	0.150	**0.406**
	Stress and poor coping	0.104	0.242	**0.582**
	Poor parental management	0.273	**0.734**	0.206
	Lack of personal/social support	0.314	**0.609**	0.193
	Community disorganization	**0.482**	0.390	0.145
Individual	Negative attitudes	**0.638**	0.219	0.418
	Risk-taking/impulsivity	0.491	0.145	**0.535**
	Substance use difficulties	**0.606**	0.196	0.097
	Anger management problems	0.368	0.232	**0.623**
	Low empathy/remorse	0.265	0.141	**0.522**
	Attention-deficit/hyperactivity disorder	0.281	0.198	**0.488**
	Poor compliance	0.493	0.232	**0.493**
	Low interest/commitment to school or work	0.440	0.161	**0.434**

Bold values represent the factor loadings.

We used the Czech standardized SAVRY version (49). Validity was calculated from a sample of 80 participants aged 12–18 (mean = 15.35, standard deviation = 1.93), including 43 girls and 37 boys (49). The correlation coefficients for individual groups of factors and overall risk, as carried out by two evaluators using Cronbach’s alpha, were 0.807 violent and 0.933 non-violent, respectively. The AUC representing the classification efficiency of the overall risk reached a high value of 0.865. Therefore, this parameter can be considered a suitable predictor of future violence (49).

#### 2.2.2. HSPQ personality questionnaire

The High School Personality Questionnaire (HSPQ) is used worldwide as a simple tool applicable in schools and forensic or clinical settings (50–52). The HSPQ is widely used in Czechia, as in several other states, with updated population norms (53–55). This inventory, developed for adolescents aged 12–18, measures personality factors and other characteristics important in predicting and understanding social, clinical, and school behaviors (50, 56). We used the Czech version (57) of the original high-school personality questionnaire (50). This self-report inventory contains 142 dimensions measuring 14 personality traits (sociability, intelligence, emotional stability, dominance, cheerfulness, conformity, boldness, sensitivity, withdrawal, apprehension, self-sufficiency, self-discipline, and tension), in which 13 of the 14 factors measured personality traits and the remaining one measured general mental ability (intelligence) (24). On average, the questionnaire took 35 min to complete (24).

### 2.3. Statistics

The Mann-Whitney *U* test was used to compare numerical variables between the two groups. For statistical calculations, we converted the SAVRY assessment of the low, medium, and high risks of violence of all 24 SAVRY risk items to numerical values (0, 1, and 2, respectively). Exploratory factor analysis with varimax rotation and the maximum likelihood method were used to determine the factors of the items (24). IBM SPSS Statistics 25 software was used for all analyses.

### 2.4. Ethics

This study was approved by the Bohnice Psychiatric Hospital Ethics Committee. The study participants and the responsible adults provided informed consent to participate. We emphasized anonymity in processing the data and reporting the findings. The Bohnice Psychiatric Hospital Law Department supervised the project (24).

## 3. Results

### 3.1. Sample description

The study sample comprised 175 men and 226 women. There were 151 participants with violence in their personal histories and 250 non-violent participants. The median of the sample was 16 years (mean = 15.4 years, standard deviation = 1.69). Most (27, 4%) adolescents were 16 years old (*N* = 110), and 12-year-olds (*N* = 30) formed 7.5% of the sample. 44 boys and 43 girls had a history of one or two acts of violence. Moreover, 37 boys and 37 girls committed three or more violent acts. Most participants were from diagnostic institutions (33%), 20% of participants were clients of an outpatient mental health facility, and 16% were hospitalized in a mental health facility. 13 percent of data were gathered from schools, 11% from children’s homes, and 7% from social care facilities (24).

### 3.2. SAVRY factor analysis

Factor analysis with varimax rotation was used to determine the item factors. The KMO test was 0.929, and Bartlett’s test of sphericity was significant (χ^2^ = 4490,529, df = 276, *p*-value < 0.001). Meeting these assumptions allowed the item structure of the exploratory factor analysis to be assessed. For maximum likelihood, this method was used for factor extraction. The analysis confirmed that the items in this part of the questionnaire were explained by three factors, which explained 45.0% of the variability. The three internal factors were the first (SAVRY items 1, 2, 3, 4, 5, 11, 16, 17, 19), the second (items 6, 7, 8, 9, 14, 15), and the third (items 10, 12, 13, 18, 20, 21, 22, 23, 24) factors (see Table 1).

### 3.3. Violence risk factors prevalence

Table 2 shows the SAVRY item assessment of the whole sample and subgroups according to violence within personal history. The group of perpetrators of violence showed higher risk factor scores than the non-violent adolescents did (24). The most frequent violence risk factor was S2 History of non-violent offending among violent adolescents (median 3, SD 0,641) compared to non-violent adolescents (median 1, SD 0,770).

**TABLE 2:** Risk and protective prevalence due to Structured Assessment of Violence Risk in Youth (SAVRY) scale in violent and non-violent adolescents.

	Risk factor presence in violent and non-violent adolescents								
SAVRY risk items	History of violence (*n* = 151)				Without history of violence (*n* = 250)				*P*-value Mann-Whitney *U* test
	Median	Mean	Std. dev.	*N*	Median	Mean	Std. dev.	*N*	
S1 history of violence	2.00	2.41	0.545	151	1.00	1.05	0.265	250	<0.001
S2 history of non-violent offending	3.00	2.54	0.641	151	1.00	1.56	0.770	250	<0.001
S3 early initiation of violence	1.00	1.60	0.665	151	1.00	1.03	0.208	250	<0.001
S4 past supervision/intervention failures	1.00	1.66	0.824	151	1.00	1.21	0.557	250	<0.001
S5 history of self-harm or suicide attempts	2.00	1.75	0.800	151	1.00	1.45	0.688	250	<0.001
S6 exposure to violence in the home	2.00	2.16	0.888	151	1.00	1.62	0.829	250	<0.001
S7 childhood history of maltreatment	2.00	2.04	0.855	151	1.00	1.63	0.817	250	<0.001
S8 parental/caregiver criminality	2.00	2.03	0.867	151	1.00	1.51	0.772	250	<0.001
S9 early caregiver disruption	3.00	2.46	0.690	151	2.00	1.82	0.849	250	<0.001
S10 poor school achievement	2.00	2.17	0.746	151	1.00	1.67	0.785	250	<0.001
S11 peer delinquency	2.00	2.13	0.797	151	1.00	1.42	0.661	250	<0.001
S12 peer rejection	2.00	1.75	0.800	151	1.00	1.51	0.696	250	<0.001
S13 stress and poor coping	2.00	2.05	0.835	151	1.00	1.60	0.733	250	<0.001
S14 poor parental management	3.00	2.68	0.582	151	2.00	2.07	0.840	250	<0.001
S15 lack of personal/social support	2.00	2.22	0.738	151	2.00	1.74	0.746	250	<0.001
S16 community disorganization	2.00	1.77	0.734	151	1.00	1.29	0.514	250	<0.001
S17 negative attitudes	2.00	2.01	0.730	151	1.00	1.29	0.520	250	<0.001
S18 risk-taking/impulsivity	2.00	2.09	0.783	151	1.00	1.42	0.674	250	<0.001
S19 substance use difficulties	2.00	1.83	0.844	151	1.00	1.20	0.518	249	<0.001
S20 anger management problems	2.00	2.28	0.750	151	1.00	1.53	0.701	250	<0.001
S21 low empathy/remorse	2.00	1.86	0.849	151	1.00	1.35	0.618	250	<0.001
S22 attention deficit/hyperactivity difficulties	2.00	1.93	0.825	151	1.00	1.46	0.640	250	<0.001
S23 poor compliance	2.00	2.19	0.647	151	2.00	1.59	0.647	250	<0.001
S24 low interest/commitment to school	2.00	2.05	0.746	151	1.00	1.45	0.620	250	<0.001

### 3.4. Violence protective factor prevalence

The most frequently occurring protective factor was the item P3 Strong Attachments and Bonds (79.8%). Contrarily, a minor common factor was P6’s resilient personality. The presence of individual factors in the examined groups is shown in Table 3. Analysis of the prevalence of protective factors in violent and non-violent adolescents (Table 3) calculated using the Mann-Whitney test showed that the presence of a protective factor significantly reduced the risk of violence. The results also showed that the prevalence of violence differed among all protective factors (24).

**TABLE 3:** Violence protective factor prevalence in violent and non-violent adolescents.

Protective factor		Violence in personal history		*P*-value Mann-Whitney	Violence risk		*P*-value Mann-Whitney
		Yes	No		Yes	No	
P1 prosocial involvement	Median	37.00	46.00	<0.001	2.00	2.00	<0.001
	Mean	38.12	46.14		1.68	2.11	
	SD	10.943	9.801		0.731	0.645	
	*N*	267	134		267	134	
P2 strong social support	Median	31.50	46.00	<0.001	1.00	2.00	<0.001
	Mean	34.69	46.20		1.38	2.22	
	SD	10.268	9.061		0.621	0.583	
	*N*	188	213		188	213	
P3 strong attachments and bonds	Median	39.00	47.00	<0.001	2.00	2.00	<0.001
	Mean	39.15	47.33		1.72	2.23	
	SD	11.082	9.259		0.727	0.597	
	*N*	320	81		320	81	
P4 positive attitude toward intervention and authority	Median	33.00	49.00	<0.001	1.00	2.00	<0.001
	Mean	33.67	48.34		1.38	2.29	
	SD	8.318	8.683		0.535	0.617	
	*N*	206	195		206	195	
P5 strong commitment to school and work	Median	29.00	47.00	<0.001	1.00	2.00	<0.001
	Mean	32.35	46.48		1.34	2.15	
	SD	8.552	9.017		0.547	0.655	
	*N*	161	240		161	240	
P6 resilient personality	Median	30.00	46.00	<0.001	1.00	2.00	<0.001
	Mean	32.82	45.73		1.41	2.08	
	SD	8.897	9.555		0.601	0.686	
	*N*	153	248		153	248	

### 3.5. Prevalence of SAVRY protective factors according to gender and history of violence

As the incidence of risk factors varies by sex, we present a protective factor analysis on the dependence on this criterion (Table 4). These factors were more prevalent in girls and boys who did not commit violence than in those with violence in their personal histories (24). The strongest protective factor was P3, Strong attachment and bonds across gender or a history of violence. In general, the highest values of the protective factors were shown for boys with no history of violence; conversely, these numbers were the lowest for girls with a history of violence. Furthermore, boys and girls with a non-violent history showed similar values of protective factors; meanwhile, regarding those with a violent history, these were lower for girls than for boys.

**TABLE 4:** Prevalence of Structured Assessment of Violence Risk in Youth (SAVRY) protective factors according to gender and history of violence.

	Violence in personal history (*n* = 151)		Without violence in personal history (*n* = 250)		Total	Absolute number
	Boys	Girls	Boys	Girls		
P1 prosocial involvement	64.2%	52.9%	80.9%	65.4%	66.6%	267
P2 strong social support	43.2%	14.3%	67.0%	51.3%	46.9%	188
P3 strong attachment and bonds	77.8%	71.4%	81.9%	83.3%	79.8%	320
P4 positive attitude toward intervention and authority	32.1%	17.1%	69.1%	66.0%	51.4%	206
P5 strong commitment to school	21.0%	12.9%	56.4%	52.6%	40.1%	161
P6 resilient personality traits	28.4%	15.7%	60.6%	39.7%	38.2%	153
Total	81	70	94	156	401	

### 3.6. SAVRY scale and HSPQ personality questionnaire items relation

The Mann-Whitney test was used to compare the two independent and dependent groups, which were formed based on the SAVRY questionnaire and in terms of the HSPQ factors, to determine whether any personality traits are typical for violent and non-violent adolescents. In Table 5, significant differences are shown in bold. There was a significant difference between Medium risk and High risk g in HSPQ A, C, Q2, and Q4 (*p*-values < 0.05). The SAVRY medium-risk group had lower ratings in Q2 and Q4 and higher ratings in A and C than the high-risk group. The protective factor prosocial involvement was significantly related to several factors of the HSPQ personality questionnaire. Specifically, these were A. Immediacy, C. Emotional stability, and Q2. Self-sufficiency, and Q4. Tension. Protective factor: Strong social support relates to factors A. Immediacy, B. High crystalline intelligence, D. Excitability, E. Dominance, F. Cheerfulness, G. Conformity, I. Sensitivity, and Q4. Tension. Strong Attachments and Bonds were significantly related only to Factor I. Sensitivity. Attitudes toward interventions and authorities were significantly related to 11 factors in the HSPQ questionnaire. Specifically, these were B. High intelligence, C. Stability, D. Excitability, E. Dominance, F. Cheerfulness, G. Conformity, I. Sensitivity, J. Withdrawal, and Q2. Self-sufficiency, Q3. Self-discipline and Q4 Tension (24). Strong commitment to school and work was significantly related to intelligence, dominance, cheerfulness, conformity, sensitivity, withdrawal, and Q2. Self-sufficiency, Q3. Self-discipline, and Q4. Tension. Resilience as a personality trait was related to the factors B. Intelligence, C. Stability, F. Cheerfulness, G. Conformity, O. Apprehension, Q.2 Self-sufficiency, Q3. Self-discipline, and Q4. Tension.

**TABLE 5:** High school personality questionnaire (HSPQ) personality questionnaire items differences between two groups.

P1 prosocial involvement	Medium risk group (*n* = 254)			High risk group (*n* = 128)			*P*-value Mann-Whitney
	Median	Mean	Std. deviation	Median	Mean	Std. deviation	
HSPQ A	12.00	11.36	3.125	10.00	9.52	3.289	<0.001
C	9.00	9.04	3.296	8.00	7.93	3.605	0.002
Q2	7.00	7.54	3.208	8.00	8.42	3.176	0.021
Q4	10.00	9.52	3.081	11.00	10.57	3.623	0.003
**P2 strong social support**	**Medium risk group (*n* = 181)**			**High risk group (*n* = 201)**			***P*-value Mann-Whitney**
	**Median**	**Mean**	**Std. deviation**	**Median**	**Mean**	**Std. deviation**	
HSPQ A	11.00	11.16	3.208	11.00	10.37	3.331	0.024
B	7.00	6.82	2.168	6.00	6.08	2.135	<0.001
D	10.00	9.83	3.101	11.00	10.79	3.258	0.003
E	9.00	9.59	3.042	11.00	10.81	2.803	<0.001
F	9.00	8.61	3.655	10.00	10.11	3.700	<0.001
G	11.00	10.94	3.400	10.00	10.05	3.339	0.012
I	12.00	11.81	3.557	11.00	11.14	3.296	0.034
Q4	9.00	8.94	3.256	11.00	10.71	3.129	<0.001
**P3 strong attachment and bonds**	**Medium risk group (*n* = 305)**			**High risk group (*n* = 77)**			***P*-value Mann-Whitney**
	**Median**	**Mean**	**Std. deviation**	**Median**	**Mean**	**Std. deviation**	
HSPQ I	12.00	11.66	3.498	10.00	10.66	3.059	0.025
**P4 positive attitude toward intervention and authority**	**1 (*n* = 200)**			**2 (*n* = 182)**			***P*-value Mann-Whitney**
	**Median**	**Mean**	**Std. deviation**	**Median**	**Mean**	**Std. deviation**	
HSPQ B	7.00	6.89	2.110	6.00	5.94	2.152	<0.001
C	9.00	9.00	3.382	8.00	8.30	3.470	0.031
D	10.00	9.87	3.096	11.00	10.86	3.274	0.002
E	9.50	9.60	2.999	11.00	10.92	2.802	<0.001
F	9.00	8.25	3.633	11.00	10.66	3.465	<0.001
G	11.00	11.13	3.219	10.00	9.75	3.439	<0.001
I	12.00	12.22	3.444	11.00	10.63	3.232	<0.001
J	10.00	9.64	2.595	9.00	9.16	2.768	0.037
Q2	7.00	7.38	3.193	8.00	8.34	3.182	0.004
Q3	11.00	10.30	3.652	9.00	9.25	3.241	0.001
Q4	9.00	8.92	3.182	11.00	10.91	3.128	<0.001
**P5 strong commitment to school and work**	**Medium risk group (*n* = 157)**			**High risk group (*n* = 225)**			***P*-value Mann-Whitney**
	**Median**	**Mean**	**Std. deviation**	**Median**	**Mean**	**Std. deviation**	
HSPQ B	7.00	7.12	1.972	6.00	5.96	2.193	<0.001
E	9.00	9.57	3.157	11.00	10.68	2.763	0.001
F	9.00	8.45	3.804	10.00	10.06	3.575	<0.001
G	11.00	11.30	3.300	10.00	9.89	3.343	<0.001
I	12.00	12.04	3.360	11.00	11.05	3.434	0.009
J	10.00	9.76	2.784	9.00	9.17	2.594	0.040
Q2	7.00	7.48	3.244	8.00	8.08	3.187	0.046
Q3	10.00	10.29	3.389	10.00	9.46	3.538	0.033
Q4	9.00	9.06	3.308	11.00	10.43	3.193	<0.001
**P6 resilient personality**	**Medium risk group (*n* = 149)**			**High risk group (*n* = 233)**			***P*-value Mann-Whitney**
	**Median**	**Mean**	**Std. deviation**	**Median**	**Mean**	**Std. deviation**	
HSPQ B	7.00	7.13	1.974	6.00	5.99	2.193	<0.001
C	10.00	9.49	3.370	8.00	8.14	3.383	<0.001
F	9.00	8.62	3.459	10.00	9.90	3.849	<0.001
G	11.00	11.01	3.120	10.00	10.13	3.520	0.013
O	11.00	11.52	3.521	12.00	12.24	3.936	0.045
Q2	7.00	7.23	3.325	8.00	8.22	3.096	0.005
Q3	11.00	10.31	3.491	10.00	9.48	3.469	0.029
Q4	9.00	9.11	3.347	11.00	10.36	3.192	<0.001

## 4. Discussion

The current study focused on the relationship between individual protective factors (items of the SAVRY scale) and HSPQ personality questionnaire characteristics. Considering our first aim, we can conclude that our sample’s risk and protective factor levels were similar to those of other studies (45, 48, 58). Non-violent adolescents in the current study had higher values for all six protective factors, adding to the evidence of the predictive validity of protective factors in violence manifestation (24, 38, 59). In addition, the results allowed us to appropriately target interventions. Girls scored orders of magnitude less than boys did in some categories; therefore, it is necessary to consider whether to use different approaches in clinical practice toward one or the other group of adolescents.

Considering our second aim, ascertaining if violence risk and protective factors were positively or negatively related to violence within personal history we found that these factors were more prevalent in girls and boys who did not commit violence than in those with violence in their personal histories. Generally, the most significant difference for girls was between violent and non-violent groups, with girls scoring lower than boys. Moreover, the most significant difference for boys was in resilient personality; thus, this factor seems to make the biggest difference between violent and non-violent boys, while for girls, it has little significance. From a clinical perspective, addressing the risk of violent adolescent behavior while supporting protective factors is essential. It remains challenging to influence risk factors because they are impossible to address directly (e.g., family socioeconomic situation, community environment, or an individual’s cognitive capacity) within intervention programs. Protective factors could be seen as opposites to risk factors, that is, the same variable but on the other end of the continuum. Another view is to consider them as separate variables that explain why some individuals with a high incidence of risk factors do not commit violence (60). Violence risk depends on protective and risk-factor interactions (24, 37, 40, 61).

The third aim of our study was identification of underlying constructs within the SAVRY tool. We identified three intrinsic SAVRY factors using factor analysis in the current study. The first relates to an individual’s active participation in dissocial behavior (substance use and peer delinquency). The second factor is assimilation, that is, denotable how the individual interacts with the environment, reflecting family functioning (maltreatment, parental management, social support). The third factor is a type of maladaptation, that is, poor adaptation to the environment (e.g., impulsivity, anger management, and poor coping). Previous SAVRY factor analyses found two factors (History of Violence/Dysregulation and Social Support) (47); one (contextual/social subscale) (62); and five factors (Antisocial Behavior, Family Functioning, Personality traits, Social Support, and Treatability) (42). Compared with our findings, this five factors structure demonstrates that our three factors overlap in Dissocial behavior with Antisocial behavior in items (1–5), with factor 2 (family functioning)–items S6–S9, and factor 4 (social support). These factors combined create the assimilation factor. Our Maladaptation factors seem to cover personality traits and treatability factors (24). We consider the perception of all 24 SAVRY items, as underlined by the three main dimensions, as beneficial in recognizing the main therapeutic focus or direction. That is, whether there is an overall antisocial dimension in each adolescent, where firm boundaries and rules are a necessity, or whether we support the dimension of assimilation and the functioning of the family system. If maladaptation is predominant, then working with impulsivity and anger management is necessary. However, in our findings, there was a cross-loading across three factors: history of non-violent offending, poor school achievement, and community disorganization. We consider Factor 1, Dissocial behavior, as the most important factor for non-violent offending and community disorganization because the given individual’s tendency for dissocial behavior is responsible for committing violence or non-violent offenses. Their activities, motives, motivation, and personality traits for committing violent acts are essential. Nevertheless, the second factor is also important in those items because external conditions also modify the probability of committing violence, such as external control or social environment. How the environment shapes the individual (e.g., parental style) is also essential. In Poor school achievement, it is predominantly maladaptive, disruptive behavior that raises concerns and warrants precautions such as facility placement, not the assimilation difficulties of the individual. Thus, it could be attributed to our sample specifics.

Our last aim was to explore, whether HSPQ factors relate to the SAVRY protective factors. We found a quite strong relationship between the protective factors of the SAVRY tool and the personality questionnaire dimensions. HSPQ diagnostics can help identify key therapeutically targetable personality traits or dimensions related to protective factors that are indirectly related to violence. In the HSPQ, a low score in factor B Intelligence, D Excitability, G Conformity, Q3 Self-discipline, or a high score on D factor Dominance or D Excitability is linked to violent, delinquent, or antisocial behavior (24, 34, 57). By identifying the critical personality factors, clinicians, social workers, and psychotherapists can consider appropriate treatment approaches and therapeutic program enrollment. The rationale for studying the relationship between HSPQ and SAVRY tools is that this widely used clinical tool relates to the SAVRY and is essential for clinical decision-making and ascertaining whether and how SAVRY assessments might help target therapeutic interventions and planning in specific clinical cases.

In our findings, the protective factor of prosocial involvement was significantly positively related to several factors in the HSPQ personality questionnaire. It is beneficial to focus therapeutically on reducing high intrapsychic tension, which is significantly associated with violence (63) and self-harm (64) in the adolescent population. Training in empathy and prosocial behavior opposes the above-mentioned inner SAVRY social behavior dimension and has been proven to reduce verbal aggression levels (65) and bullying (66).

In working with adolescents to build strong social support, it is essential to focus on reducing intrapsychic tension (24). Another therapeutic focus is anger and conflict management, which are significantly related to violence in adolescents (8, 64). A lack of self-concern and low self-esteem are helpful constructs for predicting adolescent violence (24, 29).

The strong attachment and bond factors relate only to Factor I. Sensitivity (24). Family social climate characteristics affect the HSPQ domains (67). The Cambridge Study in Delinquent Development findings show that adult crime can be predicted in childhood, suggesting that early intervention could prevent various problems of maladaptation and difficulty in adulthood (68). A close relationship with parents also promotes non-violent developmental trajectories toward social rejection at school and uses aggression to achieve social goals in interpersonal relationships (18, 23, 66). A positive relationship with at least one parent encourages social learning because early deprivation may adversely affect brain development and neuronal functioning, which are significant in regulating violent behavior (69). A Czech study showed that adolescents benefit from positive emotional relationships within the family and strong or medium educational management (70). In addition, stable positive emotional relationships are not limited to early childhood or family; a good bond with other reference people can protect against violent behavior (71).

Similar therapeutic work would also be appropriate if adolescents were to support positive SAVRY item attitudes toward interventions that are widely related to HSPQ factors. These attitudes are also important protective factors in SAPROF (5, 7, 72). Building a solid therapeutic relationship is essential (73) and working with negative attitudes is necessary. This is underscored by the fact that adolescents’ negative attitudes toward authorities are well-known risk factors for violence; meanwhile, positive attitudes have a protective effect (8, 24, 40).

To target a strong commitment to school and work, it will also be necessary to focus on therapy to reduce the risk of violence, minimize intrapsychic tension, and be closed and vigilant, which is part of the individualistic restraint factor (24). Good school achievement, a positive relationship with the school environment, motivation to reach higher education, support, and supervision by teachers, clear rules, and other positive features within a school, such as a class climate, can be strong protective factors against youth violence (36).

The last protective factor of the SAVRY is resilience as a personality trait. It will be essential to focus on reducing pressure and peer rejection (24), a risk factor for adolescent violence (8, 18). Moreover, emotion regulation ability is a significant predictor of resilience in adolescents (30). Thus, emotion-focused coping strategies are resilience-enhancing determinants. Resilient adolescents simultaneously use emotion-focused and problem-solving strategies. Coping strategies can be divided into behavioral and cognitive strategies (74). Therefore, it is crucial to focus therapy on responding to unpleasant/risky situations by concentrating on cognition to promote resilience (24).

This study had several strengths and limitations. This study combined the newly adopted SAVRY tool with a personality questionnaire. This shows how the SAVRY tool and the HSPQ self-report inventory could help identify key personality traits related to violence risk in youth. Professionals can target specific factors in psychotherapy using the SAVRY and HSPQ tools. The uniqueness of this study includes its large sample size, and enrolling male and female juveniles.

Regarding further limitations, three raters with different clinical experiences with the tools collected the data, possibly implying systematic differences in data collection. However, we aimed to reduce these influences by training raters in the SAVRY method evaluation and calculating the inter-rater reliability within the pilot study. Moreover, the sample was unbalanced, with data from 226 girls and 175 boys (of which 75 and 81 were violent, respectively). Furthermore, the sample age composition was unbalanced: participants aged 12, 13, and 18 were less represented than those aged 14, 15, and 16 (24). Another limitation is that we do not know the exact number of participants who were offered and declined participation in the study.

Furthermore, the study did not include participants with below average and lower levels of cognitive abilities who would be unable to self-reflect sufficiently to complete the personality questionnaire. Those participants could pose a high risk of violence, as low cognitive abilities are a prominent risk factor for violence; however, they did not meet the study criteria (35). In addition, the results may be affected by teenagers’ motivation to complete the HSPQ authentically. Self-report bias may have occurred in this test because most of the information cannot be verified independently. In addition to the limitations of the SAVRY tool, a large proportion of high-risk juvenile offenders are violent, non-recidivists (75). Therefore, the predictive accuracy of the SAVRY would improve if research could identify which factors (or combinations of factors) better characterize violent recidivists (24).

## 5. Conclusion

Our results showed that HSPQ diagnostics could be helpful in clinically targeting the personality-based aspects of violence risk and protective factors. SAVRY factor analysis confirms dissocial behavior as an essential factor, which, together with problematic assimilation and maladaptive strategies, constitutes the major basis of violence risk manifestation. HSPQ diagnostic results help to focus on intrapsychic tension, peer rejection, and anxiety within the clinical setting or youth intervention programs. It is also essential to foster positive attitudes toward authority, prosocial behavior, and attitudes toward school. From this perspective, we can infer that less effective intervention programs for juvenile offenders intimidate them and run in highly security-oriented facilities with restrictive environments based on bans and penalties. Nonetheless, effective interventions for adolescents would include the following factors: good relations between convicted children and facility staff; perception of direct care staff as prosocial role models; positive peer group pressure; individualized approach to intervention/therapeutic programs; programs and activities that adolescents develop appropriately; setting boundaries and expectations; functional contact with the family.

Jointly assessing and addressing protective and risk factors are crucial for evaluating and designing appropriate interventions for violent juveniles. Pointing out an individual’s strengths in treatment progress is highly desirable because it supports well-formulated decisions concerning treatment phases. Focusing on the strengths of young people and the healthy aspects of their surroundings promotes positive communication between them, their relatives and caregivers, and healthcare professionals. Altogether, it increases treatment motivation for patients and their families and rewards professionals for therapeutic work.

## Data availability statement

The raw data supporting the conclusions of this article will be made available by the authors, without undue reservation.

## Ethics statement

The studies involving human participants were reviewed and approved by the Bohnice Psychiatric Hospital Ethics Committee. Written informed consent to participate in this study was provided by the participants’ legal guardian/next of kin.

## Author contributions

PS: conception and design of the project, analysis and interpretation of data, and manuscript drafting and revision. MP: analysis and interpretation of data, manuscript drafting, revision, and final preparation. KF: interpretation of data and manuscript drafting, and revision. DP, DA, MA, and EK: manuscript drafting and revision. HB: data analysis. AM: data analysis and manuscript revision. IG: data collection and manuscript revision. All authors contributed to the manuscript and approved the submitted version.

## References

[1] de Vries Robbé M de Vogel V. A European perspective on risk assessment tools. In: Goethals K editor. *Forensic Psychiatry and Psychology in Europe.* Cham: Springer International Publishing (2018). p. 249–66.

[2] Douglas K Guy L Reeves K Weir J. *HCR-20 Violence Risk Assessment Scheme: Overview and Annotated Bibliography.* (2005). Available online at: https://escholarship.umassmed.edu/cgi/viewcontent.cgi?article=1362&context=psych_cmhsr (accessed October 13, 2019).

[3] Fazel S Singh J Doll H Grann M. Use of risk assessment instruments to predict violence and antisocial behaviour in 73 samples involving 24 827 people: systematic review and meta-analysis. *BMJ.* (2012) 345:e4692. 10.1136/bmj.e4692 22833604 PMC3404183

[4] Ramesh T Igoumenou A Vazquez Montes M Fazel S. Use of risk assessment instruments to predict violence in forensic psychiatric hospitals: a systematic review and meta-analysis. *Eur Psychiatry.* (2018) 52:47–53. 10.1016/j.eurpsy.2018.02.007 29626758 PMC6020743

[5] de Vries Robbé M de Vogel V de Spa E. Protective factors for violence risk in forensic psychiatric patients: a retrospective validation study of the SAPROF. *Int J Forensic Ment Health.* (2011) 10:178–86. 10.1186/s12991-018-0175-5 29422940 PMC5791203

[6] de Vries Robbé M de Vogel V Koster K Bogaerts S. Assessing protective factors for sexually violent offending with the SAPROF. *Sex Abuse.* (2015) 27:51–70. 25210106 10.1177/1079063214550168

[7] Páv M Vňuková M Ptáček R. *SAPROF: Strukturované Posouzení Protektivních Faktorù Při Posuzování Rizika Násilného Chování.* Praha: Ministerstvo zdravotnictví ČR (2020). p. 84.

[8] Borum R Lodewijks H Bartel P Forth A. The structured assessment of violence risk in youth (SAVRY). In: Otto RK Douglas KS editors. *Handbook of Violence Risk Assessment.* Milton Park: Routledge (2020). p. 63–79.

[9] Broulíková H Winkler P Páv M Kondrátová L. Costs of mental health services in czechia: facilitating an evidence-based reform of psychiatric care. *Appl Health Econ Health Policy.* (2020) 18:287–98. 10.1007/s40258-019-00501-7 31347015

[10] Cohen M Piquero A Jennings W. Studying the costs of crime across offender trajectories. *Criminol Public Policy.* (2010) 9:279–305.

[11] Páv M Vňuková M Sebalo I. Factors affecting length of inpatient forensic stay: retrospective study from Czechia. *Front Psychiatry.* (2022) 13:825615. 10.3389/fpsyt.2022.825615 35599778 PMC9114463

[12] Steinberg L Morris A. Adolescent development. *J Cogn Educ Psychol.* (2001) 2:55–87.

[13] Eccles J Midgley C Wigfield A Buchanan C. Development during adolescence: the impact of stage–environment fit on young adolescents’ experiences in schools and in families (1993). In: Notterman JM editor. *The Evolution of Psychology: Fifty Years of the American Psychologist.* Washington, DC: American Psychological Association (1997). p. 475–501. 10.1037//0003-066x.48.2.90 8442578

[14] Haynie D Silver E Teasdale B. Neighborhood characteristics, peer networks, and adolescent violence. *J Quant Criminol.* (2006) 22:147–69.

[15] Crick N Dodge K. Social information-processing mechanisms in reactive and proactive aggression. *Child Dev.* (1996) 67:993–1002. 8706540

[16] Juríčková V Linhartová P Adámek P Nichtová A Figueroa J Páv M Behavioral inhibition in neutral and emotional contexts in acutely violent patients with schizophrenia spectrum disorders. *Curr Psychol.* (2022) 2022:1–9.

[17] Nichtová A Volavka J Vevera J Pøíhodová K Juríèková V Klemsová A Deconstructing violence in acutely exacerbating psychotic patients. *CNS Spectr.* (2021) 26:643–7. 10.1017/S1092852920001601 32641184

[18] Fraser M. Aggressive behavior in childhood and early adolescence: an ecological-developmental perspective on youth violence. *Soc Work.* (1996) 41:347–61. 8669001

[19] Holt S Buckley H Whelan S. The impact of exposure to domestic violence on children and young people: a review of the literature. *Child Abuse Negl.* (2008) 32:797–810. 10.1016/j.chiabu.2008.02.004 18752848

[20] Perry B. The neurodevelopmental impact of violence in childhood. In: Schetky D Benedek EP editors. *Textbook of Child and Adolescent Forensic Psychiatry.* Washington, DC: American Psychiatric Press, Inc (2001). p. 221–38.

[21] Bacchini D Affuso G Aquilar S. Multiple forms and settings of exposure to violence and values: unique and interactive relationships with antisocial behavior in adolescence. *J Interpers Violence.* (2015) 30:3065–88. 10.1177/0886260514554421 25392380

[22] Bevan E Higgins D. Is domestic violence learned? The contribution of five forms of child maltreatment to men’s violence and adjustment. *J Fam Viol.* (2002) 17:223–45.

[23] Hoeve M Dubas J Eichelsheim V van der Laan P Smeenk W Gerris J. The relationship between parenting and delinquency: a meta-analysis. *J Abnorm Child Psychol.* (2009) 37:749–75. 19263213 10.1007/s10802-009-9310-8 PMC2708328

[24] Skřivánková P. *Selected Psychological Characteristics in Violence Risk Assessment in Youth in the Czech Republic.* Prague: Charles University (2022).

[25] Burt S Pearson A Carroll S Klump K Neiderhiser J. Child antisocial behavior is more environmental in origin in disadvantaged neighborhoods: evidence across residents’ perceptions and geographic scales in two samples. *J Abnorm Child Psychol.* (2019) 48:265–76. 10.1007/s10802-019-00587-6 31642028 PMC6980760

[26] Sellbom M Wygant DB Drislane LE. Elucidating the construct validity of the psychopathic personality inventory triarchic scales. *J Pers Assess.* (2015) 97:374–81. 10.1080/00223891.2014.962654 25325407

[27] Geffner R Conradi L Geis K Brenda Aranda M. Conducting child custody evaluations in the context of family violence allegations: practical techniques and suggestions for ethical practice. *J Child Custody Res Issues Pract.* (2009) 6:189–218.

[28] Johnson J Cohen P Smailes E Kasen S Oldham J Skodol A Adolescent personality disorders associated with violence and criminal behavior during adolescence and early adulthood. *Am J Psychiatry.* (2000) 157:1406–12. 10964855 10.1176/appi.ajp.157.9.1406

[29] Sutherland I Shepherd J. A personality-based model of adolescent violence. *Br J Criminol.* (2002) 42:433–41. 10.1080/15374410801955888 18470778

[30] Mestre J Núñez-Lozano J Gómez-Molinero R Zayas A Guil R. Emotion regulation ability and resilience in a sample of adolescents from a suburban area. *Front Psychol.* (2017) 8:1980. 10.3389/fpsyg.2017.01980 29180978 PMC5693909

[31] Naragon-Gainey K McMahon T Chacko T. The structure of common emotion regulation strategies: a meta-analytic examination. *Psychol Bull.* (2017) 143:384–427. 10.1037/bul0000093 28301202

[32] Nestor P. Mental disorder and violence: personality dimensions and clinical features. *Am J Psychiatry.* (2002) 159:1973–8. 12450942 10.1176/appi.ajp.159.12.1973

[33] Juarros-Basterretxea J Herrero J Escoda-Menéndez P Rodríguez-Díaz F. Cluster B personality traits and psychological intimate partner violence: considering the mediational role of alcohol. (2020) 37:N1566–87. Available online at: https://journals.sagepub.com/doi/abs/10.1177/0886260520922351 (accessed October 10, 2022). 10.1177/0886260520922351 32538293

[34] Carlota A. Filipino female juvenile delinquents: an exploratory study of their level of intelligence, personality, and attitudes towards the self and selected social figures. *Philipp J Psychol.* (1982) 15–16:3–27.

[35] Jacob L Haro J Koyanagi A. Association between intelligence quotient and violence perpetration in the English general population. *Psychol Med.* (2019) 49:1316–23. 10.1017/S0033291718001939 30058504

[36] Lösel F Farrington D. Direct protective and buffering protective factors in the development of youth violence. *Am J Prev Med.* (2012) 43:S8–23. 22789961 10.1016/j.amepre.2012.04.029

[37] de Vries Robbé M Geers M Stapel M Hilterman E de Vogel V. *SAPROF Youth Version: Guidelines for the Assessment of Protective Factors for Violence Risk in Juveniles.* Utrecht: Van der Hoeven Kliniek (2015).

[38] Kleeven A de Vries Robbé M Mulder E Popma A. Risk assessment in juvenile and young adult offenders: predictive validity of the SAVRY and SAPROF-YV. *Assessment.* (2022) 29:181–97. 10.1177/1073191120959740 32964720 PMC8796163

[39] Burghart M de Ruiter C Hynes SE Krishnan N Levtova Y Uyar A. The structured assessment of protective factors for violence risk (SAPROF): a meta-analysis of its predictive and incremental validity. *Psychol Assess.* (2022). 10.1037/pas0001184 36227302

[40] Páv M Skřivánková P Vňuková M Ptáček RV. Hodnocení rizika násilného jednání. *Ceska Slov Psychiatr.* (2020) 116:66–73.

[41] Catchpole R Gretton H. The predictive validity of risk assessment with violent young offenders: a 1-year examination of criminal outcome. *Crim Justice Behav.* (2016) 30:688–708.

[42] Hilterman E Nicholls T van Nieuwenhuizen C. Predictive validity of risk assessments in juvenile offenders: comparing the SAVRY, PCL:YV, and YLS/CMI with unstructured clinical assessments. *Assessment.* (2014) 21:324–39. 10.1177/1073191113498113 23921605

[43] Vincent G Guy L Gershenson B Mccabe P. Does risk assessment make a difference? Results of implementing the SAVRY in juvenile probation. *Behav Sci Law.* (2012) 30:384–405. 10.1002/bsl.2014 22745028

[44] Koh L Day A Klettke B Daffern M Chu C. Youth violence assessment instruments: are they sensitive to change and are changes related to recidivism? *Psychol Crime Law.* (2021) 28:416–33. 10.1034/j.1600-0447.106.s412.10.x 12072126

[45] Soderstrom M Childs K Frick P. The role of protective factors in the predictive accuracy of the structured assessment of violence risk in youth (SAVRY). *Youth Violence Juv Justice.* (2019) 18:78–95.

[46] Fabrigar L MacCallum R Wegener D Strahan E. Evaluating the use of exploratory factor analysis in psychological research. *Psychol Methods.* (1999) 4:272–99.

[47] Sijtsema J Kretschmer T van Os T. The structured assessment of violence risk in youth in a large community sample of young adult males and females: the TRAILS study. *Psychol Assess.* (2015) 27:669–77. 10.1037/a0038520 25558967

[48] Hilterman E Bongers I Nicholls T van Nieuwenhuizen C. Identifying gender specific risk/need areas for male and female juvenile offenders: factor analyses with the structured assessment of violence risk in youth (SAVRY). *Law Hum Behav.* (2016) 40:82–96. 10.1037/lhb0000158 26390056

[49] Skřivánková P. *Strukturované Hodnocení Rizika Násilí u Dospívajících.* Praha: Hogrefe – Testcentrum (2020).

[50] Cattell R Cattell M. *High School Personality Questionnaire.* Champaign, IL: Institute for personality and ability testing (1968).

[51] Srivastava G. Development of norms for Hindi adaptation of junior-senior HSPQ (1968 version). *Asian J Psychol Educ.* (1982) 9:43–7.

[52] Cattell R Gibbons B. Personality factor structure of the combined guilford and cattell personality questionnaires. *J Pers Soc Psychol.* (1968) 9:107–20. 10.1037/h0025724 5667433

[53] Cattell R. *HSPQ Cuestionario de Personalidad Para Adolescentes (12-18 años).* Madrid: TEA Ediciones (1983).

[54] Dolejš M. *Updating of the Population Standards for High School Personality Questionnaire (HSPQ).* Olomouc: STARFOS (2014).

[55] Sherman JL Krug SE Birenbaum M. Checking the reliability and validity of HSPQ profiles. *J Pers Assess.* (1979) 43:644–7. 10.1207/s15327752jpa4306_15 16366994

[56] Cattell R Coan R Beloff H. A re-examination of personality structure in late childhood, and development of the high school personality questionnaire. *J Exp Educ.* (2015) 27:73–88.

[57] Balcar K. *Osobnostní Dotazník Pro Mládež HSPQ (II. Přepracované Vydání).* Bratislava: Psychodiagnostické a didaktické testy (1986).

[58] Lodewijks H Doreleijers T de Ruiter C Borum R. Predictive validity of the structured assessment of violence risk in youth (SAVRY) during residential treatment. *Int J Law Psychiatry.* (2008) 31:263–71. 10.1016/j.ijlp.2008.04.009 18508122

[59] Zhou J Witt K Cao X Chen C Wang X. Predicting reoffending using the structured assessment of violence risk in youth (SAVRY): a 5-year follow-up study of male juvenile offenders in Hunan Province, China. *PLoS One.* (2017) 12:e0169251. 10.1371/journal.pone.0169251 28076443 PMC5226674

[60] Herrenkohl T Kosterman R Mason W Hawkins J. Youth violence trajectories and proximal characteristics of intimate partner violence. *Violence Vict.* (2007) 22:259–74. 10.1891/088667007780842793 17619633

[61] de Vogel V de Vries Robbé M de Ruiter C Bouman Y. Assessing protective factors in forensic psychiatric practice: introducing the SAPROF. *Int J Forensic Ment Health.* (2011) 10:171–7.

[62] Childs K Frick P Gottlieb K. Sex differences in the measurement invariance and factors that influence structured judgments of risk using the structured assessment of violence risk in youth (SAVRY). *Youth Violence Juv Justice.* (2014) 14:76–92.

[63] Langhinrichsen-Rohling J Neidig P. Violent backgrounds of economically disadvantaged youth: risk factors for perpetrating violence? *J Fam Violence.* (1995) 10:379–97.

[64] Laye-Gindhu A Schonert-Reichl K. Nonsuicidal self-harm among community adolescents: understanding the “whats” and “whys” of self-harm. *J Youth Adolesc.* (2005) 34:447–57.

[65] Caprara G Kanacri B Gerbino M Zuffianň A Alessandri G Vecchio G Positive effects of promoting prosocial behavior in early adolescence. *Int J Behav Dev.* (2014) 38:386–96.

[66] Waasdorp T Bradshaw C Leaf P. The impact of schoolwide positive behavioral interventions and supports on bullying and peer rejection: a randomized controlled effectiveness trial. *Arch Pediatr Adolesc Med.* (2012) 166:149–56. 10.1001/archpediatrics.2011.755 22312173

[67] Forman SG Forman BD. Family environment and its relation to adolescent personality factors. *J Pers Assess.* (1981) 45:163–7. 10.1207/s15327752jpa4502_11 16370733

[68] Zara G Farrington D. Childhood and adolescent predictors of late onset criminal careers. *J Youth Adolesc.* (2009) 38:287–300. 10.1007/s10964-008-9350-3 19636745

[69] Kraemer G. Social attachment, brain function, aggression and violence. In: Raine A Brennan PA Farrington DP Mednick SA editors. *Biosocial Bases of Violence.* Boston, MA: Springer (1997). p. 207–29.

[70] Jan Č. *Rozvíjení Osobnosti a Zpùsob Výchovy. ISV.* (1996).

[71] Werner E Ruth S. *Journeys From Childhood to Midlife: Praha, Risk, Resilience, and Recovery.* Ithaca, NY: Cornell University Press (2001).

[72] Páv M Sebalo I Vňuková M Pabiánová Š Málová V Hollý M Predicting discharge from long-term forensic treatment: patients characteristics, protective factors, needs and treatment-related factors study in the Czechia. *J Forensic Psychiatry Psychol.* (2022) 33:89–111. 10.1080/14789949.2022.2027995

[73] Lambert M Barley D. Research summary on the therapeutic relationship and psychotherapy outcome. *Psychotherapy.* (2001) 38:357–61.

[74] Garnefski N Kraaij V Spinhoven P. Negative life events, cognitive emotion regulation and emotional problems. *Pers Individ Diff.* (2001) 30:1311–27.

[75] Chu C Goh M Chong D. The predictive validity of savry ratings for assessing youth offenders in Singapore: a comparison with YLS/CMI ratings. *Crim Justice Behav.* (2016) 43:793–810. 10.1177/0093854815616842 27231403 PMC4843087

